# Artificial Intelligence for Patient Safety and Surgical Education in Neurosurgery

**DOI:** 10.31662/jmaj.2024-0141

**Published:** 2024-08-30

**Authors:** Taku Sugiyama, Hiroyuki Sugimori, Minghui Tang, Miki Fujimura

**Affiliations:** 1 Department of Neurosurgery, Hokkaido University Graduate School of Medicine, Sapporo, Japan; 2 Medical AI Research and Development Center, Hokkaido University Hospital, Sapporo, Japan; 3 Department of Biomedical Science and Engineering, Faculty of Health Sciences, Hokkaido University, Sapporo, Japan; 4 Department of Diagnostic Imaging, Hokkaido University Faculty of Medicine and Graduate School of Medicine, Sapporo, Japan

**Keywords:** AI, computer vision, deep learning, machine learning, microsurgery, surgical skill

## Abstract

Neurosurgery has evolved alongside technological innovations; however, these advances have also introduced greater complexity into clinical practice. Neurosurgery remains a demanding and high-risk field that requires a broad range of skills. Artificial intelligence (AI) has immense potential in neurosurgery given its ability to rapidly analyze large volumes of clinical data generated in modern clinical environments. An expanding body of literature has demonstrated that AI enhances various aspects of neurosurgery, including diagnostics, prognostication, decision-making, data management, education, and clinical studies. AI applications are expected to reduce medical errors and costs, broaden healthcare accessibility, and ultimately boost patient safety and surgical education. Nevertheless, AI application in neurosurgery remains practically limited because of several challenges, such as the diversity and volume of clinical training data collection, concerns regarding data quality, algorithmic bias, transparency (explainability and interpretability), ethical issues, and regulatory implications. To comprehensively discuss the potential benefits, future directions, and limitations of AI in neurosurgery, this review examined recent studies on AI technology and its applications in this field, focusing on intraoperative decision support and surgical education.

## Introduction

Advances in neurosurgery have closely paralleled technological developments, which have significantly improved patient outcomes in contemporary neurosurgery practices ^(1)^. However, these advancements have increased complexity by incorporating multiple technologies and fostering multidisciplinary approaches ^(2), (3)^.

In modern operating rooms, neurosurgeons are equipped with an array of surgical devices and monitors and collaborate with diverse teams, including anesthesiologists, technicians, and nurses. The role of neurosurgeons requires not only a profound understanding of various neurological disorders, including demographics, etiology, genetics, pathophysiology, diagnostic techniques, and prognosis, but also expertise in complex neuroanatomy, diverse surgical procedures, advanced psychomotor skills, and leadership in communication with other staff members. Neurosurgery remains a demanding field that requires mastery of cognitive, decision-making, and technical surgical skills through extensive, intensive, and prolonged training ^(4)^. Neurosurgeons must assimilate, retain, analyze, and interpret vast, dynamic, and complex datasets and tasks that increasingly challenge human capabilities. Moreover, when treating patients in the acute phase, quick decision-making is required in many clinical settings. Reducing door-to-intervention time during emergency procedures often compromises the time available for physicians to process enormous amounts of clinical data.

Given the aforementioned challenges and critical significance of the central nervous system, the incidence of adverse events in neurosurgical procedures might be notably higher than that in other medical disciplines, significantly affecting patients’ quality of life ^(5), (6), (7)^. The literature indicates that approximately 25% of these events are attributable to preventable technical errors, underscoring the need for systematic measures to mitigate such medical errors and ensure patient care that yields satisfactory outcomes.

Among the most expected recent advancements in neurosurgery, artificial intelligence (AI) applications include machine learning (ML), natural language processing (NLP), computer vision (CV), and artificial neural networks (ANNs) ^(8)^. The potential of AI in neurosurgery is substantial and is supported by its ability to rapidly analyze large volumes of clinical data generated in contemporary settings, which surpass human capabilities. Specialized algorithms can be applied to diverse multimodal clinical data sources, including alphanumeric electronic health charts, laboratory testing, radiological images, electrophysiological examinations, and clinical data from photographs and videos. A previous study indicated that AI can improve various aspects of neurosurgery, including diagnostics, prognostication, clinical decision-making, data management, education, and clinical research ^(8)^. AI applications can reduce medical errors, decrease costs, broaden healthcare availability, and enhance patient safety and surgical education.

However, the practical application of AI in neurosurgery remains limited because of several challenges, such as the diversity and volume of clinical training data collection, concerns regarding data quality, algorithmic bias, transparency (explainability and interpretability), ethical issues, and regulatory implications. This review comprehensively examines recent studies on AI technology and its applications to better understand its potential benefits, future perspectives, and limitations in neurosurgery.

## AI for Diagnosing and Classifying Neurological Disorders

As exemplified by the computer-assisted diagnosis of gliomas ^(9)^, AI has been frequently used to aid diagnostic approaches. The capability of AI to rapidly process enormous amounts of anatomical, morphological, and connectivity information from imaging data makes computer-assisted diagnosis one of the most promising areas for AI applications, assisting neuroradiologists and neurosurgeons, and reducing human labor and costs. Gliomas have been successfully detected and differentiated from other central nervous system pathologies ^(10)^. Tumor grading and prediction of isocitrate dehydrogenase mutation ^(11)^, 1p19q co-deletion ^(12)^, O-6-methylguanine-DNA-methyltransferase promoter status ^(13)^, and overall survival rates have been achieved using predictive AI models ^(9), (14)^. Combining radiomics data with clinical variables has facilitated the development of various ML models that can reliably differentiate the molecular subgroups of brain tumors ^(15)^.

Similarly, for the detection of intracranial aneurysms, deep learning (DL) algorithms have been found to be more efficient than radiologists ^(16)^. The prediction of aneurysm stability has also been investigated using prediction models ^(17), (18)^. Applications of AI in the fields of functional and spinal surgeries have led to achievements in electroencephalogram analysis, seizure categorization, epilepsy subtype classification, automated detection of seizures in videos, and detection of lesions compressing the spinal cord ^(19), (20), (21)^.

AI applications in aiding the diagnosis of radiological imaging have been well documented in the literature and have reached the stage of broad clinical application. However, to effectively train, validate, and test diagnostic AI systems, well-annotated datasets that include imaging data and tissue samples are required. These datasets are costly and time-consuming. Additionally, many intracranial diseases, including glioma, are relatively rare, making the acquisition of large volumes of clinical data a significant challenge ^(9)^.

## AI Application in the Outcome and Risk Prediction of Neurosurgical Procedures

Predicting outcomes, postoperative complications, and adverse events remains a critical focus for neurosurgeons. A previous study revealed that the gradient boosting ML algorithm outperformed traditional statistical methods in predicting early postoperative complications following intracranial tumor surgery ^(22)^. Another study successfully used conditional ML algorithms and inference tree analysis to predict perioperative transfusion needs among adult patients undergoing spinal deformity surgery ^(23)^. ML technologies have been extensively tested for various neurosurgical scenarios, including predicting survival rates following brain metastasis resection ^(24)^, seizure-free outcomes after anterior temporal lobectomy ^(25)^, short-term mortality and adverse events after spinal surgery ^(26)^, the likelihood of aneurysm rupture, the outcomes and incidence of delayed cerebral ischemia following subarachnoid hemorrhage ^(27), (28)^, and clinical outcomes following flow diversion stent treatments for intracranial aneurysms ^(29)^.

Although these results are promising, a recent systematic review revealed that the majority of these studies were single-center investigations, with only 15% of them conducting external validation using an independent cohort of patients ^(30)^. This lack of transparency in the underlying logic of ML models makes most clinicians hesitant to trust their predictions provided by ML models ^(31)^. Similar to traditional statistical methods, such as logistic regression, explainability, and interpretability, future AI models should address these challenges. Another inherent limitation of these studies was that most risk predictions were provided preoperatively. However, most risky intraoperative events occur unexpectedly, and in practical clinical situations, the surgical team must manage these events reactively by relying on clinical assessment. Therefore, real-time feedback in the operating room is critical for clinical decision support using AI is required.

## AI Application in Intraoperative Decision Support during Neurosurgery

The primary objective of neurosurgery is maximal resection or eradication of pathological lesions while preserving neurological function. AI has the potential to enhance surgical performance and reduce the risk of medical errors during the intraoperative phase of neurosurgery. Shen et al. and Hollon et al. implemented deep convolutional neural networks in conjunction with indocyanine green fluorescence imaging and Raman spectroscopy to facilitate near-real-time intraoperative glioma diagnosis ^(32), (33)^. These methodologies enable tumor diagnosis within 3 min, which is a significant improvement over the traditional rapid frozen section pathological diagnosis, which typically requires up to 30 min. Similarly, Cakmakci et al. used a random forest model in conjunction with high-resolution magic angle spinning nuclear magnetic resonance spectroscopy to accurately determine the metabolic profile of the resected tissue intraoperatively, identify residual tumorous tissue in the excision cavity, and guide surgeons toward maximal resection ^(34)^. Qiao et al. used automated DL analysis for intraoperative visually evoked potentials during endoscopic transsphenoidal surgery, thereby preventing optic nerve injury and reducing labor-intensive work by electrophysiologists ^(35)^.

The utility of AI extends to surgical planning, particularly in determining the appropriate surgical approach, and it holds promise for improving outcomes and minimizing neurological sequelae. Dundar et al. proposed a surgical route planning algorithm that combines heuristics with Q-learning, a reinforcement learning-based AI technique, to identify optimal skull entry points and routes for minimally invasive tumor removal ^(36)^. Given that intraoperative brain shifts pose a significant challenge during cranial surgery, Tonutti et al. used ANNs and support vector regression in combination with the finite element method (FEM) to predict the deformation at each node in a brain tumor mesh model. They reported on-screen positional errors of <0.4-0.5 and 0.2 mm compared with an average error of 1.4 mm for other deformation models ^(37)^. These results highlight that ML algorithms can be applied to enhance FEM-based biomechanical simulations of anatomical structures, allowing for real-time responses ^(38)^.

Automated operative workflow analysis using CV technologies has also been applied to surgical procedures, such as endoscopic transsphenoidal surgery. The ML model was able to distinguish the various steps and techniques for excision of pituitary adenoma with high accuracy, even with significant variability in the training data ^(39)^. Disruptions in procedural flow during surgery can lead to errors and increase the risk of complications. Common causes include teamwork or communication failures, technology and equipment malfunctions, and training-related distractions.

Currently, most surgical procedures are condensed into a single-page operative report that serves as the standard information source for all surgical disciplines. Despite the availability of surgical video recordings and data from multiple monitoring systems. However, a previous study showed that these surgical reports failed to detail nearly one-third of intraoperative adverse events. The current paradigm of “surgical data science” emphasizes the importance of evaluating both how the operation proceeds and how effectively the surgeon performed the surgery ^(40), (41)^. Given the massive amount of data, including monitoring data and video recordings, the incorporation of AI technologies into data acquisition, curation, and analysis is considered the most promising solution to this problem. The major obstacles to the widespread use of AI are as follows: (1) anatomical structures in the surgical field are often hidden under layers of other tissues or obscured by a surgeon’s hands, making it difficult to train models for detecting specific anatomical structures or surgical instruments; and (2) there is significant variation, even among expert surgeons, in how they perform surgical procedures, necessitating comprehensive datasets that encompass various surgical expertise.

## AI Application in Surgical Education and Training

Surgeon proficiency plays a pivotal role in the outcomes of surgical procedures and significantly influences adverse events, morbidity, and mortality ^(42)^. Implementing comprehensive evaluations of surgical skills provides a reliable measure that not only identifies and corrects deficiencies in surgical trainees through constructive feedback but also accredits surgeon competence in society, thereby enhancing both surgical education and patient safety ^(43)^.

Traditional assessment of surgical skills relied primarily on subjective evaluations by the surgeon. Several criteria-based scoring systems were also used in these evaluations. However, these methods are limited by the significant human and time resources required and the potential for inter-rater bias ^(43)^. Efforts have been made to develop quantifiable evaluation methodologies that address subjectivity. Notably, engineering technologies using specialized tools, such as sensors affixed to surgical instruments to measure force ^(43), (44)^ or electromagnetic trackers to monitor the movements of surgeons’ hands or instruments ^(45)^, have been developed. Recently, AI technologies have been used for data analysis to quickly provide feedback with minimal human resources ^(46), (47)^. Another innovative approach involves the use of virtual reality (VR) simulators ^(48), (49)^. These simulators are particularly effective for repeated training sessions and facilitate easy data collection, although they may not fully capture the physical characteristics of tissues and psychological pressures present in real surgical situations ^(50)^.

Although these methods facilitate an objective assessment of skill levels, their reliance on specialized equipment, such as sensors or VR simulators, limits their widespread adoption and raises concerns about their generalizability and reproducibility. In recent years, there has been a shift toward analytical methods that leverage video recordings of surgical procedures. The proposed method is gaining traction because of its applicability to a broad array of operating room environments ^(51)^. Additionally, the use of AI technologies to evaluate surgical skills via video analysis is becoming increasingly common ^(40), (52)^.

**Figure 1.**
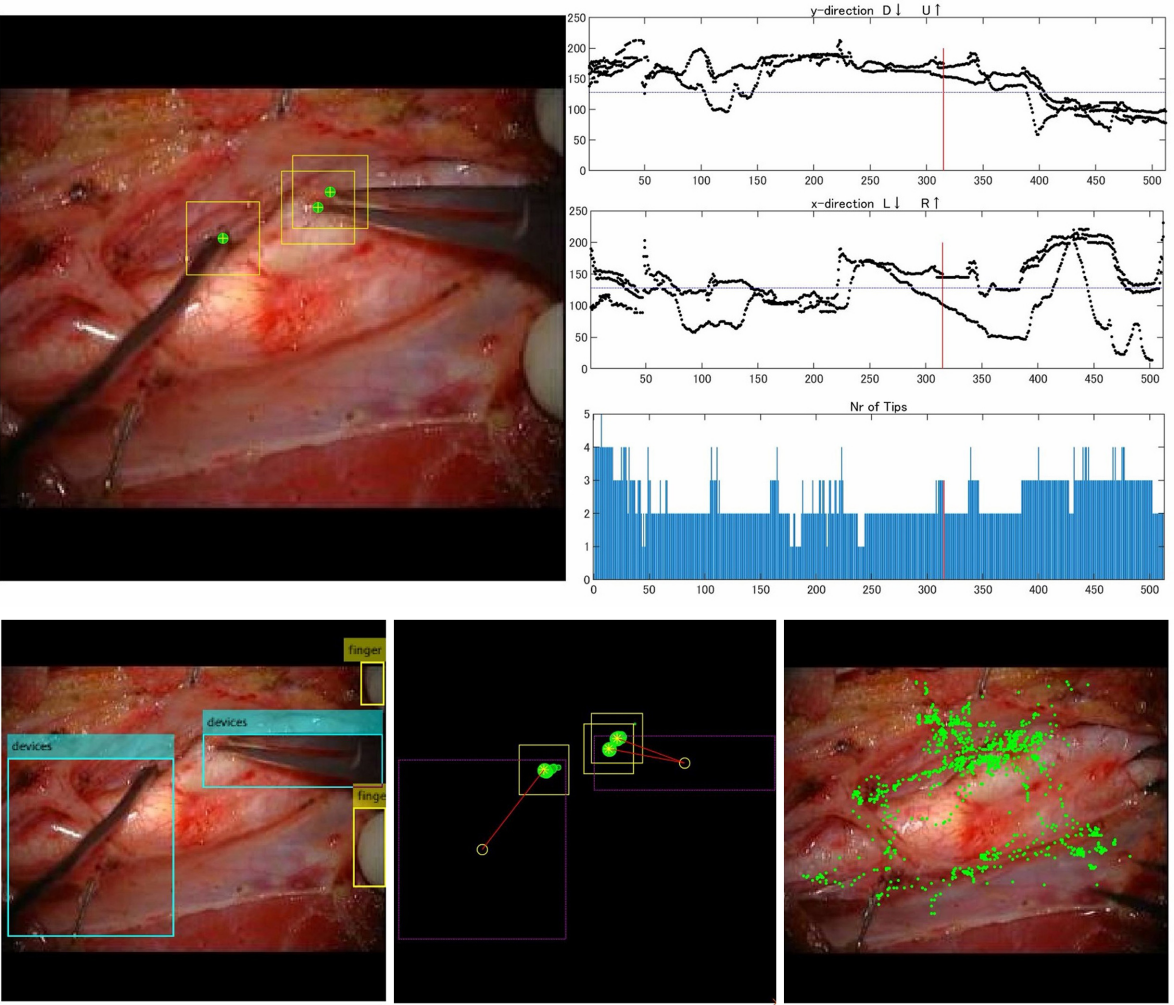
Example of the home-made automated instrument detection software for carotid endarterectomy.

**Figure 2.**
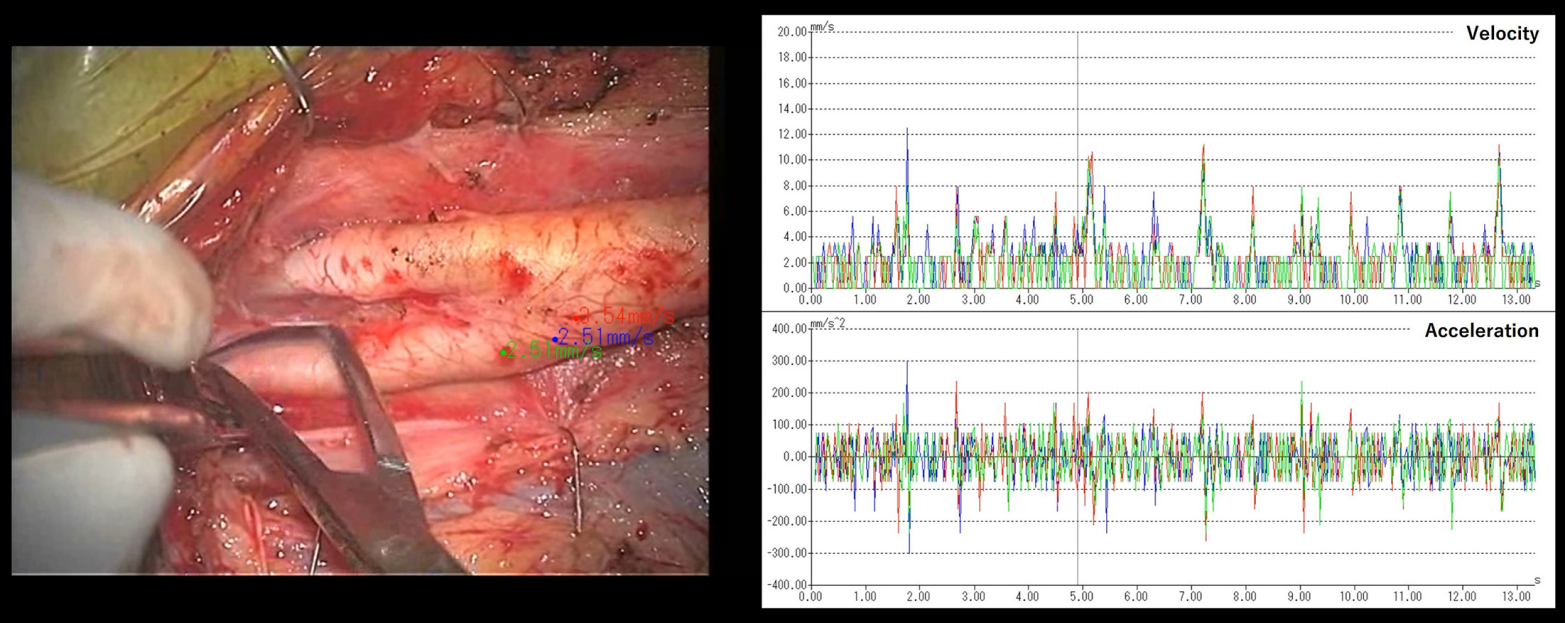
Example tissue motion analysis during carotid endarterectomy.

Pangal et al. used an automated instrument detection algorithm to calculate performance metrics for predicting overall success rates, blood loss, and hemorrhage control within <1 min of endoscopic endonasal surgery using a cadaveric internal carotid artery vascular injury simulator ^(53)^. The findings indicate that these performance metrics are more reliable predictors of surgical outcomes than the training status or prior experience of the surgeons ^(53)^. Our group also developed an automated instrument detection algorithm for carotid endarterectomy (Figure 1) ^(52)^. Additionally, we proposed a novel metric for the objective and quantitative assessment of surgical skills by focusing on tissue motion during surgical maneuvers (Figure 2). Analysis of the motion parameters (e.g., acceleration and velocity) of target tissues (carotid plaques) during carotid endarterectomy revealed that novice surgeons typically exhibit higher tissue motion parameter values than expert surgeons ^(54), (55)^. Additionally, this metric correlates with adverse events (ischemic stroke and cervical nerve injury) during surgery ^(54)^. These findings indicate that minimizing tissue movement during surgery can serve as a novel indicator of surgical skill and outcomes, emphasizing “gentleness” in tissue handling ^(54), (55)^.

**Figure 3.**
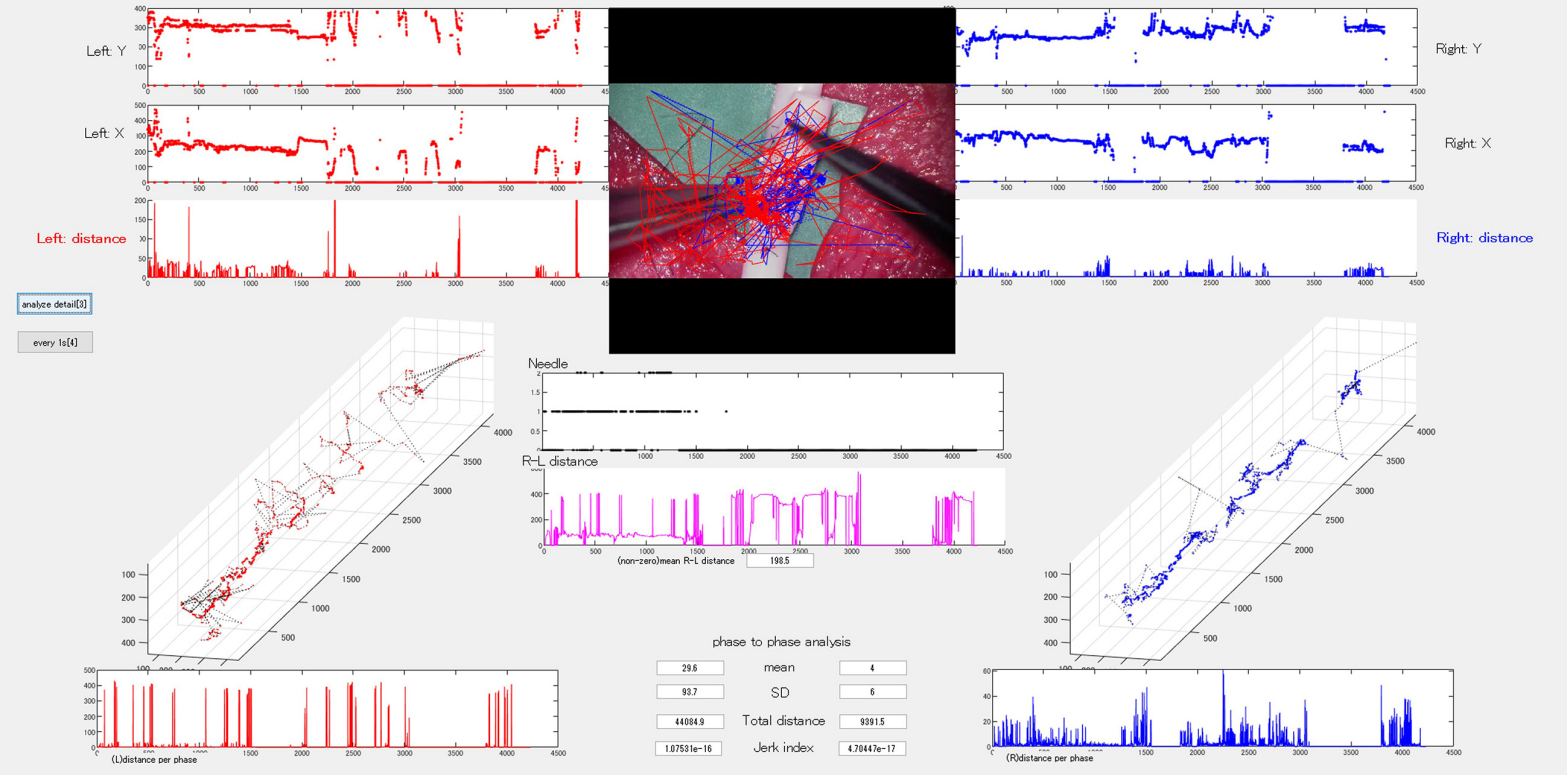
Surgical instrument tip trajectory analysis artificial intelligence model.

As microvascular procedures require specialized technical skills to handle fragile intracranial arteries, several AI applications have been reported for microvascular anastomosis training. Gonzalez-Romo et al. developed an ML-based model capable of tracking 21 hand landmarks for microvascular anastomosis simulation ^(56)^. Recently, our study group developed and used an automated instrument tip-tracking algorithm to measure motion economy (procedural time and path distance) and motion smoothness (normalized jerk index) during the task of suturing artificial blood vessels (Figure 3) ^(57)^. Similar findings have been observed in microsurgical arachnoid dissection ^(58)^.

**Figure 4.**
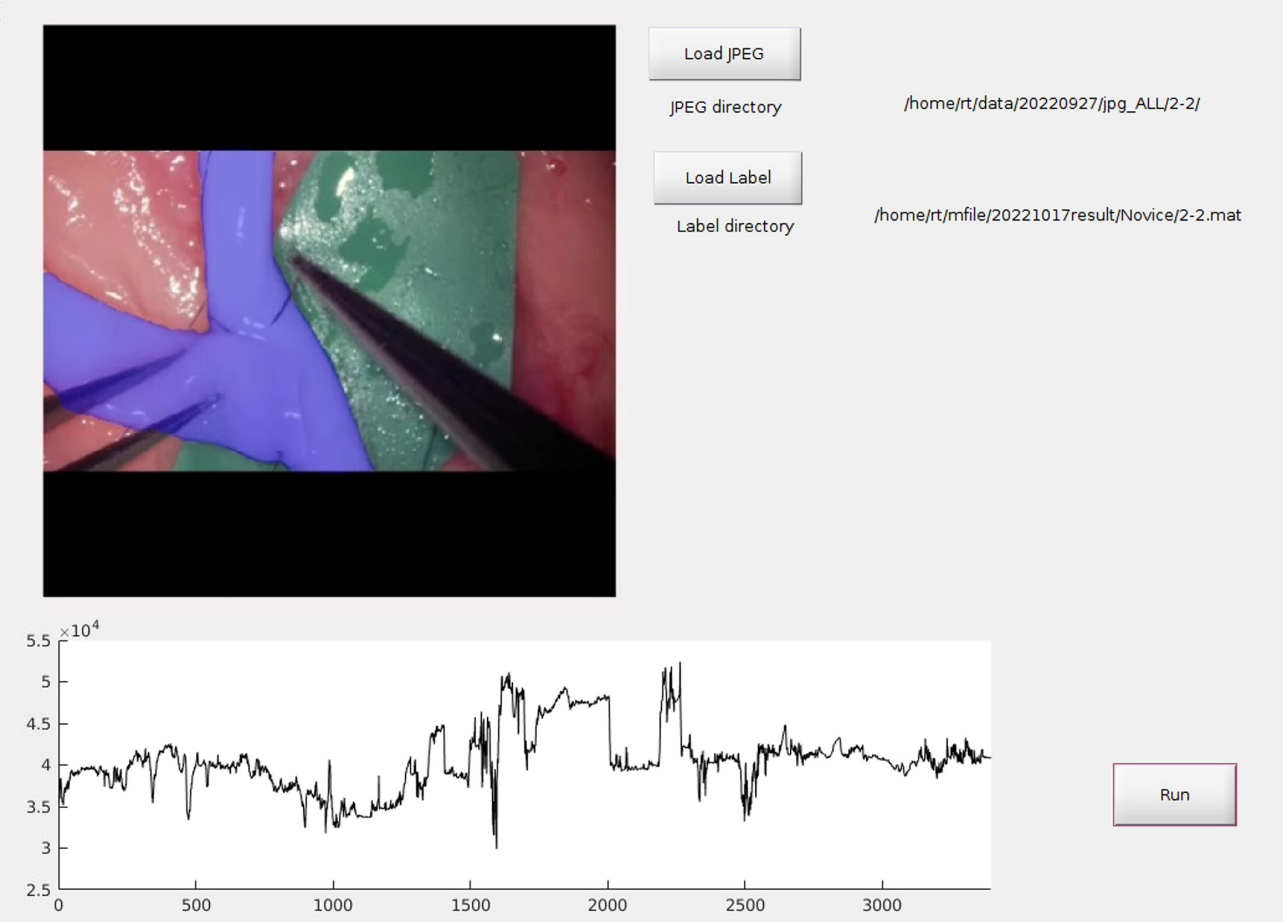
Semantic segmentation artificial intelligence model of blood vessels.

Additionally, we developed a DL-based semantic segmentation algorithm to assess changes in vessel area under the hypothesis that avoiding excessive or abrupt vessel deformation could serve as a novel metric for gentle maneuvering during microvascular anastomosis (Figure 4) ^(59)^. By applying these models, we demonstrated differences between experts and novices in terms of video parameters and provided technical challenges for trainee surgeons. These studies did not evaluate training efficiency through repeated training sessions after providing feedback on their techniques; thus, further studies are necessary to validate training efficiency and estimate learning curves.

**Figure 5.**
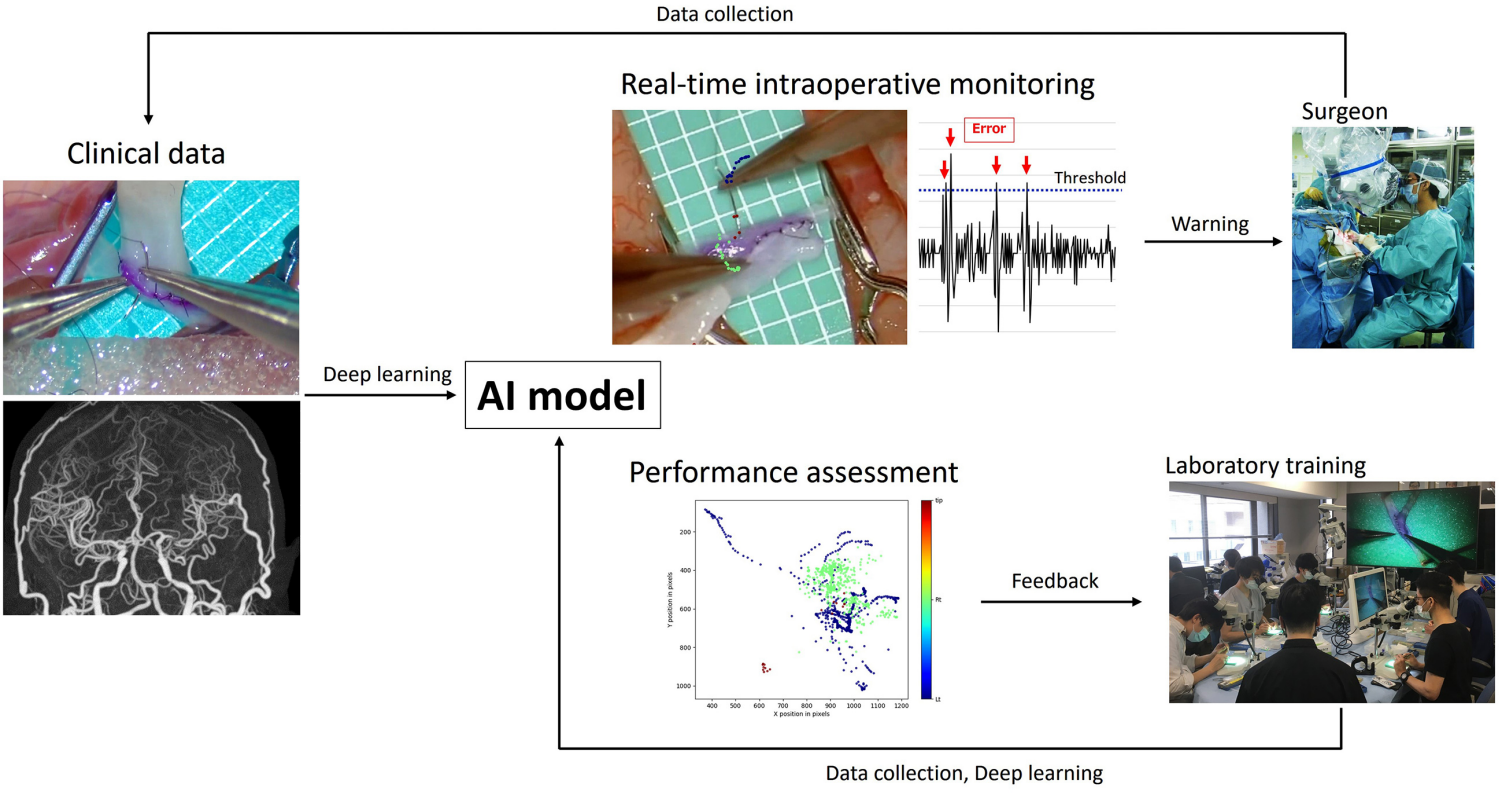
Overview of our research project on artificial intelligence (AI) for neurosurgery aimed at enhancing patient safety and improving surgical education (circulation model for AI enhancement).

A data-driven analysis of surgical performance can numerically define several surgical errors that have been proven to be correlated with intraoperative adverse events ^(44), (54), (59)^. Therefore, AI-powered real-time monitoring with warning systems should be developed and introduced in clinical practice (Figure 5). However, given the extensive variety of technical surgical nuances, further exploratory data-driven studies should be conducted to identify metrics that align surgical proficiency with patient outcomes.

## NLP Application in Clinical Practice

Large language models (LLMs) are generative AI models that produce human-like texts. Recently, their popularity has surged, with significant applications in studies, education, and clinical tasks ^(60)^. This trend extends to neurosurgery, in which LLMs are actively integrated into daily practice.

Previous investigations into the capabilities of ChatGPT (versions GPT-3.5 and GPT-4.0) and Google Bard have shown high performance in U.S. medical licensing examination- and neurosurgical board examination-style questions, demonstrating their potential utility in standardized medical testing ^(61), (62), (63)^. In neurosurgical studies and documentation, ChatGPT has been used to facilitate the accurate coding of neurosurgical procedures, highlighting its broad logistical utility ^(64)^.

In clinical applications, LLMs have shown promising diagnostic capabilities. For example, GPT-4.0 demonstrated an accuracy of up to 62% in classifying specific types of tumors during neuroradiological evaluation ^(65)^. Furthermore, LLMs have been used in treatment queries ^(66), (67)^, neurosurgical triage and consultation ^(68)^, and procedural inquiries ^(69)^, indicating their potential use as clinical and diagnostic aids. The development of neuroGPT-X has demonstrated performance on par with or superior to that of expert responses in terms of coherence, thoroughness, relevance, and accuracy ^(70)^. These applications highlight the need for this technology and its capacity to deliver prompt and readily available clinical information to patients, students, and healthcare professionals. LLMs can promote the synthesis of academic studies and their translation into clinical practice ^(10)^.

Additionally, NLP techniques are used to analyze free text in patient medical records. NLP, a subfield of AI, uses tools to encode concepts and derive meanings from unstructured text, thereby enhancing the understanding of patient data ^(71)^. Tirukumaran et al. discovered that models using NLP could predict surgical site infections in orthopedic surgery patients with a level of accuracy similar to that of manual data extraction and better than models relying solely on administrative data ^(72)^.

## Potential Risks and Challenges of AI Application in Neurosurgery

The integration of AI into neurosurgery presents significant challenges and risks that must be addressed to optimize outcomes and maintain ethical standards. One major challenge is to ensure that both patients and healthcare providers can effectively interpret and understand AI-driven models and the implications for treatment and predictive outcomes. Despite the high accuracy of their predictions, AI systems are not without errors or uncertainties, and uncritical reliance on these technologies can lead to undue dependency and potential mismanagement ^(8)^.

The complex nature of neural networks, with their multiple processing layers, often remains opaque, creating what is commonly referred to as an “algorithmic black box.” Understanding these mechanisms is crucial when using AI in clinical settings. Additionally, AI systems generally lack the versatility of human analytical skills because many clinical AI applications are designed to perform specific tasks within limited contexts. Training algorithms for data from specific institutions or patient demographics can introduce inherent biases that reflect the characteristics of the training data rather than a general patient population. Explainable AI is a promising solution to these issues ^(73), (74)^. However, the limited applicability of current AI models highlights the need for enhanced human oversight to prevent errors.

Furthermore, excessive reliance on AI can diminish the clinical expertise and decision-making capabilities of surgeons. Dependence on AI recommendations without a thorough evaluation can result in overlooked diagnoses or ineffective treatment strategies ^(8), (20)^.

Another critical concern is cybersecurity ^(20)^. AI systems are networked; thus, vulnerabilities may lead to unauthorized access, data manipulation, or disruptions during surgical procedures. The necessity for vast amounts of patient data to develop robust AI models also raises ethical concerns regarding data privacy.

A significant array of clinically relevant algorithms must be implemented. The debate over whether patient data should be recorded is ongoing; however, it is crucial to address the ethical and legal implications of potential AI-driven misdiagnoses. Moreover, clinicians require computer science training to better understand and leverage these AI systems.

## Future Perspectives

AI has already demonstrated diagnostic and prognostic accuracy comparable with that of experienced clinicians. A promising approach for enhancing clinical diagnosis is to integrate the expertise of experienced clinicians into AI algorithms. By incorporating insights from these experts, neurosurgical trainees can gain valuable guidance to develop their clinical skills.

In neurosurgery, AI can also enhance the function of surgical adjuncts. For example, semiautomated positioning of endoscopes or microscopes using predefined anatomical landmarks derived from imaging has shown potential for intracranial and spinal surgeries ^(4)^. Additionally, AI can improve image-guidance systems using preoperative or intraoperative imaging data to assist in planning surgical paths. Automated systems can further aid surgeons by providing alerts when critical structures, such as blood vessels and sensitive areas, are at risk. AI-powered neurosurgical VR simulators can efficiently and accurately assess the skills of residents and medical students.

Surgical robots that offer mechanical advantages over humans can be further improved. By combining robotic assistance with advanced sensory technologies, such as machine vision, haptic feedback, motion sensors, and automated control algorithms, human surgical capabilities can be extended beyond their current limits ^(1), (8)^. Furthermore, integrating brain-computer interfaces and AI can restore sensory and motor functions in patients with paralysis, enhance the motor abilities of healthy individuals, and drive the development of next-generation robots. Signals from the brain can be captured noninvasively using electroencephalography and processed to control robotic arms ^(4), (75)^.

## Conclusions

AI applications have the potential to significantly enhance various aspects of neurosurgical practice, including diagnostics, prognostication, decision-making, and data management. This can improve patient safety and surgical education. It is crucial to thoroughly understand and address the potential risks, limitations, and challenges associated with AI before its widespread adoption. Future clinicians must remain informed about the advancements in health care and be capable of integrating these innovations into their practices to achieve better outcomes.

## Article Information

### Conflicts of Interest

None

### Sources of Funding

This work was supported by JSPS KAKENHI grant number JP21K09091.

### Acknowledgement

We would like to thank Editage (www.editage.com) for its English language editing.

### Author Contributions

T.S. contributed to study conception and design. Data were collected and analyzed by T.S., H.S., and M.T. Drafts of the manuscript were written by T.S. All authors have read and approved the final manuscript. T.S. contributed to the grant acquisition. M.F. contributed to study supervision.

### Approval by Institutional Review Board (IRB)

This study was approved by the institutional review board of Hokkaido University Hospital, Sapporo, Japan (No. 018-0291).

### Disclaimer

Miki Fujimura is one of the Editors of JMA Journal and on the journal’s Editorial Staff. He was not involved in the editorial evaluation or decision to accept this article for publication at all.
